# Supplementary material for: Discovery and application of insertion-deletion (INDEL) polymorphisms for QTL mapping of early life-history traits in Atlantic salmon
Source: BMC Genomics. 2010 Mar 8;11:156. doi: 10.1186/1471-2164-11-156 (PMC2838853; doi:10.1186/1471-2164-11-156)
Supplement: Additional file 2 — Information on developed 76 locus single-run INDEL panel in Atlantic salmon. Information on fluorescence labeling, primer concentrations, PCR pooling and links to alignments, INDEL motifs and GENESCAN (Burge and Karlin 1997) predictions of genes/exons are available in html format. [file 1471-2164-11-156-S2.ZIP › Additionalfile2/table8417.html]

Contig Alignment

```
Base	A B C D E F G H   
1	. . . G . G . .    
2	. . . G . G . .    
3	. . . G . G . .    
4	. . . G . G . .    
5	. . . G . G . .    
6	. . . G . G . .    
7	. . . T . T . .    
8	. . . G . G . .    
9	. . . T . T . .    
10	. . . C . C . .    
11	. . . T . T . .    
12	. . . G . G . .    
13	. . . T . T . .    
14	. . . T . T . .    
15	. . . A . A . .    
16	. . . T . T . .    
17	. . . A . A . .    
18	. . . T . T . .    
19	. . . C . C . .    
20	. . . G . G . .    
21	. . . C . C . .    
22	. . . T . T . .    
23	. . . G . G . .    
24	. . . T . T . .    
25	. . . T . T . .    
26	. . . G . G . .    
27	. . . T . T . .    
28	. . . A . A . .    
29	. . . T . T . .    
30	. . . T . T . .    
31	. . . A . A . .    
32	. . . T . T . .    
33	. . . C . C . .    
34	. . . A . A . .    
35	. . . G . G . .    
36	. . . A . A . .    
37	. . . C . C . .    
38	. . . T . T . .    
39	. . . A . A . .    
40	. . . C . C . .    
41	. . . C . C . .    
42	. . . A . A . .    
43	. . . G . G . .    
44	. . . T . T . .    
45	. . . A . A . .    
46	. . . T . T . .    
47	. . . T . T . .    
48	. . . C . C . .    
49	. . . A . A . .    
50	. . . A . A . .    
51	. . . G . G . .    
52	. . . T . T . .    
53	. . . T . T . .    
54	. . . A . A . .    
55	A . . A A A . .    
56	T . . T T T . .    
57	T . . T T T . .    
58	C . . C C C . .    
59	A . A A A A . .    
60	A . A A A A . .    
61	G . G G G G . .    
62	A . A A A A . .    
63	C . C C C C . .    
64	T . T T T T . T    
65	T . T T T T . T    
66	G . G G G G . G    
67	G . G G G G . G    
68	G . G G G G . G    
69	G . G G G G . G    
70	C . C C C C . C    
71	A . A A A A . A    
72	G . G G G G . G    
73	A . A A A A . A    
74	A . A A A A . A    
75	T . T T T T . T    
76	G . G G G G . G    
77	A . A A A A . A    
78	G . G G G G . G    
79	C . C C C C . C    
80	C . C C C C . C    
81	A . A A A A . A    
82	T . T T T T . T    
83	C . C C C C . C    
84	T . T T T T . T    
85	A . A A A A . A    
86	A . A A A A . A    
87	A . A A A A . A    
88	A . A A A A . A    
89	C . C C C C . C    
90	A . A A A A . A    
91	G . G G G G . G    
92	A . A A A A . A    
93	A . A A A A . A    
94	A . A A A A . A    
95	C . C C C C . C    
96	A A A A A A . A    
97	T T T T T T . T    
98	C C C C C C . C    
99	T T T T T T . T    
100	A A A A A A . A    
101	A A A A A A . A    
102	C C C C C C . C    
103	C C C C C C . C    
104	G G G G G G . G    
105	T T T T T T . T    
106	C C C C C C . C    
107	T T T T C T . C    
108	T T T T T T . T    
109	A A A A A A . A    
110	A A A A A A . A    
111	T T T T T T . T    
112	T T T T T T . T    
113	A A A A A A . A    
114	T T T T T T . T    
115	T T T T T T . T    
116	G G G G G G . G    
117	A A A A A A . A    
118	T T T T T T . T    
119	G G G G G G . G    
120	C C C C C C . C    
121	C C C C C C . C    
122	T T T T T T . T    
123	T T T T T T . T    
124	A A A A A A . A    
125	A A A A A A . A    
126	A A A A A A . A    
127	A A A A A A . A    
128	A A A A A A . A    
129	G G G G G G . G    
130	G G G G G G . G    
131	T T T T T T . T    
132	C C C C C C . C    
133	C C C C C C . C    
134	A A A A A A . A    
135	A A A A A A . A    
136	T T T T T T . T    
137	G G G G G G . G    
138	C C C C C C . C    
139	A A A A A A . A    
140	G G G G G G . G    
141	C C C C C C . C    
142	A A A A A A . A    
143	G G G G G G . G    
144	T T T T T T . T    
145	T T T T T T . T    
146	C C C C C C . C    
147	A A A A A A . A    
148	A A A A A A . A    
149	T T T T T T . T    
150	A A A A A A . A    
151	G G G G G G . G    
152	A A A A A A . A    
153	T T T T T T . T    
154	T T T T T T . T    
155	A A A A A A . A    
156	C C C C C C . G    
157	G G G G G G . G    
158	A A A A A A . A    
159	T T T T T T . T    
160	T T T T T T . T    
161	A A A A A A . A    
162	T T T T T T . T    
163	T T T T T T . T    
164	C C C C C C . C    
165	T T T T T T . T    
166	G G G G G G . G    
167	C C C C C C . C    
168	G G G G G G . G    
169	C C C C C C . C    
170	A A A A A A . A    
171	A A A A A A . A    
172	C C C C C C . C    
173	A A A A A A . A    
174	A A A A A A . A    
175	T T T T T T . T    
176	T T T T T T . T    
177	A A A A A A . A    
178	A A A A A A . A    
179	G G G G G G . G    
180	T T T T T T . T    
181	A A A A A A . A    
182	C C C C C C . C    
183	C C C C C C . C    
184	T T T T T T . T    
185	A A A A A A . A    
186	A A A A A A . A    
187	A A A A A A . A    
188	T T T T T T . T    
189	T T T T T T . T    
190	G G G G G G . G    
191	T T T T T T . T    
192	G G G G G G . G    
193	A A A A A A . A    
194	T T T T T T . T    
195	C C C C C C . C    
196	A A A A A A . A    
197	A A A A A A . A    
198	T T T T T T . T    
199	T T T T T T . T    
200	T T T T T T . T    
201	A A A A A A . A    
202	A A A A A A . A    
203	A A A A A A . A    
204	A A A A A A . A    
205	C C C C C C . C    
206	A A A A A A . A    
207	A A A A A A . A    
208	A A A A A A . A    
209	A A A A A A . A    
210	T T T T T T . T    
211	G G G G G G . G    
212	G G G G G G . G    
213	T T T T T T . T    
214	A A A A A A . A    
215	G G G G G G . G    
216	A A A A A A . A    
217	A A A A A A . A    
218	A A A A A A . A    
219	A A A A A A . A    
220	C C C C C C . C    
221	A A A A A A . A    
222	A A A A A A . A    
223	A A A A A A . A    
224	- - - T T T . T  3	 indel  
225	- - - A A A . A  3	 indel  
226	G G G G G G . G    
227	A A A A A A . A    
228	A A A A A A . A    
229	A A A A A A . A    
230	T T T T T T . T    
231	T T T T T T . T    
232	T T T T T T . T    
233	C C C C C C . C    
234	T T T T T T . T    
235	C C C C C C . C    
236	A A A A A A . A    
237	A A A A A A . A    
238	G G G G G G . G    
239	C C C C C C . C    
240	A A A A A A . A    
241	A A A A A A . A    
242	G G G G G G . G    
243	A A A A A A . A    
244	A A A A A A . A    
245	T T T T T T . T    
246	T T T T T T . T    
247	T T T T T T . T    
248	T T T T T T . T    
249	G G G G G G . G    
250	C C C C C C . C    
251	T T T T T T . T    
252	C C C C C C . C    
253	A A A A A A . A    
254	G G G G G G . G    
255	A A A A A A . A    
256	C C C C C C . C    
257	T T T T T T . T    
258	T T T T T T . T    
259	T T T T T T . T    
260	C C C C C C . C    
261	T T T T T T . T    
262	G G G G G G . G    
263	G G G G G G . G    
264	G G G G G G . G    
265	A A A A A A . A    
266	G G G G G G . G    
267	C C C C C C . C    
268	G G G G G G . G    
269	G G G G G G . G    
270	T T T T T T . T    
271	T T T T T T . T    
272	T T T T T T . T    
273	G G G G G G . G    
274	A A A A A A . A    
275	G G G G G G . G    
276	T T T T T T . T    
277	G G G G G G . G    
278	G G G G G G . G    
279	G G G G G G . G    
280	G G G G G G . G    
281	A A A A A A . A    
282	A A A A A A . A    
283	G G G G G G . G    
284	G G G G G G . G    
285	G G G G G G . G    
286	A A A A A A . A    
287	A A A A A A . A    
288	A A A A A A . A    
289	A A A A A A . A    
290	C C C C C C . C    
291	G G G G G G . G    
292	A A A A A A . A    
293	G G G G G G . G    
294	T T T T T T . T    
295	T T T T T T . T    
296	A A A A A A . A    
297	T T T T T T . T    
298	T T T T T T . T    
299	G G G G G G . G    
300	T T T T T T . T    
301	A A A A A A . A    
302	A A A A A A . A    
303	G G G G G G . G    
304	A A G A A A . A    
305	G G G G G G . G    
306	G G G G G G . G    
307	T T T T T T . T    
308	T T T T T T . T    
309	G G G G G G . G    
310	G G G G G G . G    
311	G G G G G G . G    
312	A A A A A A . A    
313	A A A A A A . A    
314	T T T T T T . T    
315	T T T T T T . T    
316	A A A A A A . A    
317	A A A A A A . A    
318	A A A A A A . A    
319	T T T T T T . T    
320	T T T T T T . T    
321	T T T T T T . T    
322	A A A A A A . A    
323	C C C C C C . C    
324	C C C C C C . C    
325	A A A A A A . A    
326	C C C C C C . C    
327	A A A A A A . A    
328	T T T T T T . T    
329	G G G G G G . G    
330	A A A A A A . A    
331	T T T T T T . T    
332	G G G G G G . G    
333	T T T T T T . T    
334	C C C C C C . C    
335	A A A A A A . C    
336	C C C C C C . C    
337	C C C C C C . C    
338	A A A A A A . A    
339	T T T T T T . T    
340	G G G G G G G G    
341	G G G G G G G G    
342	A A A A A A A A    
343	A A A A A A A A    
344	G G G G G G G G    
345	G G G G G G G G    
346	C C C C C C C C    
347	C C C C C C C C    
348	A A A A A A A A    
349	G G G G G G G G    
350	A A A A A A A A    
351	A A A A A A A A    
352	C C C C C C C C    
353	T T T T T T T T    
354	C C C C C C C C    
355	C C C C C C C C    
356	C C C C C C C C    
357	T T T T T T T T    
358	C C C C C C C C    
359	C C C C C C C C    
360	C C C C C C C C    
361	A A A A A A A A    
362	T T T T T T T T    
363	C C C C C C C C    
364	A A A A A A A A    
365	A A A A A A A A    
366	A A A A A A A A    
367	A A A A A A A A    
368	C C C C C C C C    
369	A A A A A A A A    
370	G G G G G G G G    
371	G G G G G G G G    
372	C C C C C C C C    
373	T T T T T T T T    
374	G G G G G G G G    
375	A A A A A A A A    
376	A A A A A A A A    
377	A A A A A A A A    
378	T T T T T T T T    
379	T T T T T T T T    
380	A A A A A A A A    
381	C C C C C C C C    
382	A A A A A A A A    
383	G G G G G G G G    
384	G G G G G G G G    
385	A A A A A A A A    
386	A A A A A A A A    
387	T T T T T T T T    
388	T T T T T T T T    
389	A A A C C C A C  4	 transversion  
390	T T T T T T T T    
391	T T T T T T T T    
392	T T T T T T T T    
393	T T T T T T T T    
394	C C C C C C C C    
395	A A A A A A A A    
396	A A A A A A A A    
397	A A A A A A A A    
398	C C C C C C C C    
399	A A A A A A A A    
400	G G G G G G G G    
401	C C C C C C C C    
402	T T T T T T T T    
403	C C C C C C C C    
404	C C C C C C C C    
405	T T T T T T T T    
406	A A A A A A C A    
407	C C C C C C C C    
408	A A A A A A A A    
409	C C C C C C C C    
410	T T T T T T T T    
411	A A A A A A A A    
412	A A A A A A A A    
413	A A A A A A A A    
414	A A A A A A A A    
415	G G G G G G G G    
416	G G G G G G G G    
417	G G G G G G G G    
418	C C C C C C C C    
419	A A A A A A A A    
420	T T T T T T T T    
421	T T T T T T T T    
422	A A A A A A A A    
423	A A A A A A A A    
424	C C C C C C C C    
425	A A A A A A A A    
426	T T T T T T T T    
427	T T T T T T T T    
428	A A A A A A A A    
429	T T T T T T T T    
430	T T T T T T T T    
431	T T T T T T T T    
432	T T T T T T T T    
433	C C C C C C C C    
434	A A A A A A A A    
435	C C C C C C C C    
436	A A A A A A A A    
437	A A A A A A A A    
438	T T T T T T T T    
439	A A A A A A A A    
440	T T T T T T T T    
441	T T T T T T T T    
442	A A A A A A A A    
443	T T T T T T T T    
444	T T T T T T T T    
445	C C C C C C C C    
446	C C C C C C C C    
447	A A A A A A A A    
448	A A A A A A A A    
449	C C C C C C C C    
450	C C C C C C C C    
451	T T T T T T T T    
452	C C C C C C C C    
453	A A A A A A A A    
454	G G G G G G G G    
455	T T T T T T T T    
456	G G G G G G G G    
457	T T T T T T T T    
458	G G G G G G G G    
459	G G G G G G G G    
460	A A A A A A A A    
461	A A A A A A A A    
462	A A A A A A A A    
463	A A A A A A A A    
464	A A A A A A A A    
465	T T T T T T T T    
466	A A A A A A A A    
467	T T T T T T T T    
468	A A A A A A A A    
469	T T T T T T T T    
470	A A A A A A A A    
471	A A A A A A A A    
472	A A A A A A A A    
473	A A A A A A A A    
474	C C C C C C C C    
475	A A A A A A A C    
476	C C C C C C C C    
477	A A A A A A A A    
478	G G G G G G G G    
479	G G G G G G G G    
480	A A A A A A A A    
481	A A A A A A A A    
482	A A A A A A A A    
483	A A A A A A A A    
484	T T T T T T T T    
485	C C C C C C C C    
486	A A A A A A A A    
487	A A A A A A A A    
488	G G G G G G G G    
489	T T T T T T T T    
490	T T T T T T T T    
491	T T T T T T T T    
492	G G G G G G G G    
493	A A A A A A A A    
494	T T T T T T T T    
495	T T T T T T T T    
496	G G G G G G G G    
497	C C C C C C C C    
498	A A A A A A A C    
499	C C C C C C C C    
500	T T T T T T T T    
501	G G G G G G G G    
502	G G G G G G G G    
503	G G G G G G G G    
504	C C C C C C C C    
505	C C C C C C C C    
506	T T T T T T T T    
507	T T T T T T T T    
508	T T T T T T T T    
509	A A A A A A A A    
510	A A A A A A A A    
511	A A A A A A A A    
512	T T T T T T T T    
513	G G G G G G G G    
514	A A A A A A A A    
515	T T T T T T T T    
516	T T T T T T T T    
517	T T T T T T T T    
518	A A A A A A A A    
519	A A A A A A A A    
520	A A A A A A A A    
521	T T T T T T T T    
522	G G G G G G G G    
523	A A A A A A A A    
524	A A A A A A A A    
525	A A A A A A A A    
526	A A A A A A A A    
527	T T T T T T T T    
528	G G G G G G G G    
529	C C C C C C C C    
530	C C C C C C C C    
531	T T T T T T T T    
532	T T T T T T T T    
533	A A A A A A A A    
534	C C C C C C C C    
535	A A A A A A A A    
536	C C C C C C C C    
537	T T T T T T T T    
538	A A A A A A A A    
539	C C C C C C C C    
540	C C C C C C C C    
541	A A A A A A A A    
542	T T T T T T T T    
543	C C C C C C C C    
544	T T T T T T T T    
545	C C C C C C C C    
546	A A A A A A A A    
547	C C C C C C C C    
548	T T T T T T T T    
549	C C C C C C C C    
550	A A A A A A A A    
551	T T T T T T T T    
552	T T T T T T T T    
553	C C C C C C C C    
554	A A A A A A A A    
555	T T T T T T T T    
556	C C C C C C C C    
557	C C C C C C C C    
558	A A A A A A A A    
559	T T T T T T T T    
560	A A A A A A A A    
561	T T T T T T T T    
562	T T T T T T T T    
563	A A A A A A A A    
564	G G G G G G G G    
565	T T T T T T T T    
566	T T T T T T T T    
567	A A A A A A A A    
568	T T T T T T T T    
569	T T T T T T T T    
570	C C C C C C C C    
571	T T T T T T T T    
572	G G G G G G G G    
573	T T T T T T T T    
574	G G G G G G G G    
575	A A A A A A A A    
576	C C C C C C C C    
577	T T T T T T T T    
578	T T T T T T T T    
579	T T T T T T T T    
580	C C C C C C C C    
581	A A A A A A A A    
582	G G G G G G G G    
583	T T T T T T T T    
584	T T T T T T T T    
585	C C C C C C C C    
586	A A A A A A A A    
587	C C C C C C C C    
588	T T T T T T T T    
589	T T T T T T T T    
590	G G G G G G G G    
591	T T T T G T T G    
592	. . . T T T T T    
593	. . . T T T T T    
594	. . . T T T T T    
595	. . . T T T T T    
596	. . . T T T T T    
597	. . . T T T T T    
598	. . . T T T T T    
599	. . . C C C C C    
600	. . . - - - C -    
601	. . . A A A A A    
602	. . . T T T T T    
603	. . . G G G G G    
604	. . . T T T T T    
605	. . . C C C C C    
606	. . . C C C C C    
607	. . . T T T T T    
608	. . . G G G G G    
609	. . . C C C C C    
610	. . . T T T T T    
611	. . . G G G G G    
612	. . . C C C C C    
613	. . . T T T T T    
614	. . . T T T T T    
615	. . . T T T T T    
616	. . . T T T T T    
617	. . . T T T T T    
618	. . . G G G G G    
619	. . . G G G G G    
620	. . . G G G G G    
621	. . . T T T T T    
622	. . . C C C C C    
623	. . . A A A A A    
624	. . . T T T T T    
625	. . . A A A A A    
626	. . . T T T T T    
627	. . . T T T T T    
628	. . . T T T T T    
629	. . . T T T T T    
630	. . . T T T T T    
631	. . . C C C C C    
632	. . . A A A A A    
633	. . . A A A A A    
634	. . . C C C C C    
635	. . . A A A C C    
636	. . . C C C C C    
637	. . . C C C C C    
638	. . . A A A A A    
639	. . . A A A A A    
640	. . . A A A A A    
641	. . . G G G G G    
642	. . . A A A A A    
643	. . . A A A A A    
644	. . . T T T T T    
645	. . . G G G G G    
646	. . . A A A A A    
647	. . . A A A A A    
648	. . . T T T T T    
649	. . . T T T T T    
650	. . . C C C C C    
651	. . . A A A A A    
652	. . . A A A A A    
653	. . . A A A A A    
654	. . . C C C C C    
655	. . . T T T T T    
656	. . . G G G G G    
657	. . . A A A A A    
658	. . . C C C C C    
659	. . . A A A A A    
660	. . . G G G G G    
661	. . . T T T T T    
662	. . . T T T T T    
663	. . . T T T T T    
664	. . . A A A A A    
665	. . . T T T T T    
666	. . . A A A A A    
667	. . . G G G G G    
668	. . . G G G G G    
669	. . . C C C C C    
670	. . . T T T T T    
671	. . . A A A A A    
672	. . . T T T T T    
673	. . . A A A A A    
674	. . . A A A A A    
675	. . . T T T T T    
676	. . . C C C C C    
677	. . . T T T T T    
678	. . . G G G G G    
679	. . . T T T T T    
680	. . . T T T T T    
681	. . . C C C C C    
682	. . . T T T T T    
683	. . . G G G G G    
684	. . . C C C C C    
685	. . . C C C C C    
686	. . . A A A A A    
687	. . . A A A A A    
688	. . . A A A A A    
689	. . . A A A A A    
690	. . . G G G G G    
691	. . . T T T T T    
692	. . . T T T T T    
693	. . . A A A A A    
694	. . . A A A A A    
695	. . . A A A A A    
696	. . . T T T T T    
697	. . . A A A A A    
698	. . . A A A A A    
699	. . . A A A A A    
700	. . . A A A A A    
701	. . . C C C C C    
702	. . . T T T T T    
703	. . . T T T T T    
704	. . . T T T T T    
705	. . . T T T T T    
706	. . . C C C C C    
707	. . . A A A A A    
708	. . . A A A A A    
709	. . . C C C C C    
710	. . . G G G G G    
711	. . . C C C C C    
712	. . . T T T T T    
713	. . . C C C C C    
714	. . . . . . . C    
715	. . . . . . . G    
716	. . . . . . . A    
717	. . . . . . . C    
718	. . . . . . . A    
719	. . . . . . . G    
720	. . . . . . . C    
721	. . . . . . . C    
722	. . . . . . . C    
723	. . . . . . . C    
724	. . . . . . . A    
725	. . . . . . . T
```
